# 1-[3-(4-Chloro­phen­yl)-6-methyl-1,6-di­hydro-1,2,4,5-tetra­zin-1-yl]ethanone

**DOI:** 10.1107/S1600536810037839

**Published:** 2010-09-30

**Authors:** Feng Xu, Zhenzhen Yang, Junrong Jiang, Lei Shi

**Affiliations:** a Department of Biological & Chemical Engineering, Taizhou Vocational & Technical College, Taizhou, 318000, People’s Republic of China

## Abstract

In the title compound, C_11_H_11_ClN_4_O, the tetra­zine ring adopts a non-symmetrical boat conformation. The crystal packing exhibits relatively short inter­molecular C⋯N contacts of 3.118 (3) Å.

## Related literature

For related structures, see: Hu *et al.* (2004 ▶, 2005 ▶); Jennison *et al.* (1986 ▶); Stam *et al.* (1982 ▶); Xu *et al.* (2010 ▶); Yang *et al.* (2010 ▶). For applications of 1,2,4,5-tetra­zine derivatives, see: Sauer (1996 ▶). 
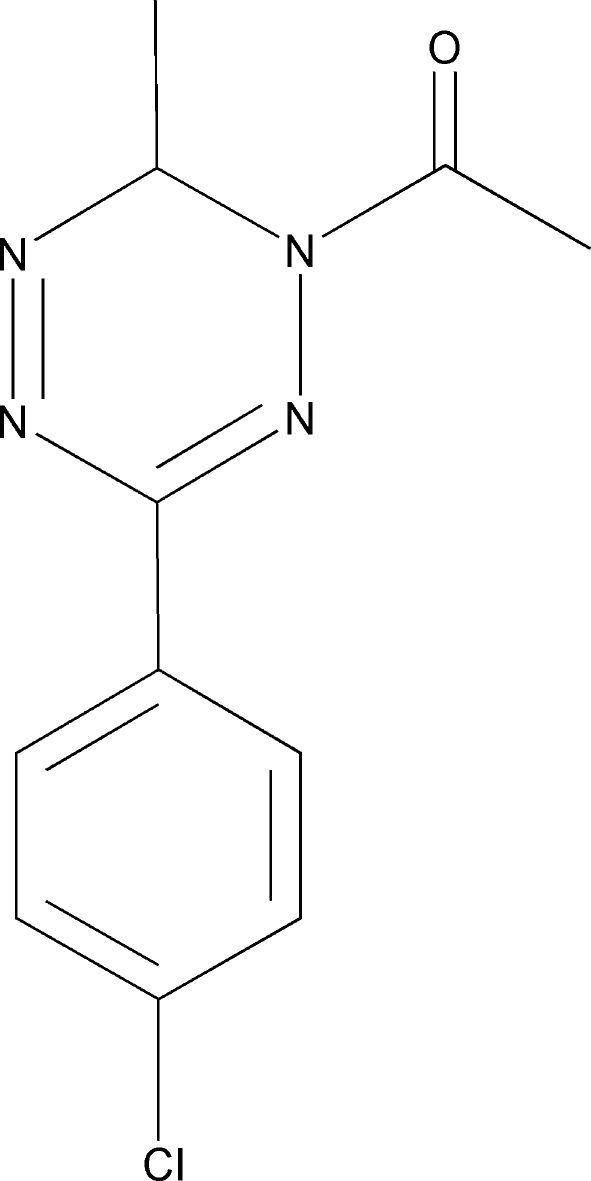


## Experimental

### 

#### Crystal data

C_11_H_11_ClN_4_O
*M* *_r_* = 250.69
Orthorhombic, 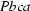

*a* = 15.165 (3) Å
*b* = 8.0452 (15) Å
*c* = 19.349 (4) Å
*V* = 2360.7 (8) Å^3^
*Z* = 8
Mo *K*α radiation
μ = 0.31 mm^−1^
*T* = 93 K
0.47 × 0.40 × 0.37 mm

#### Data collection

Rigaku AFC10/Saturn724+ diffractometer
17617 measured reflections
2705 independent reflections
2599 reflections with *I* > 2σ(*I*)
*R*_int_ = 0.038

#### Refinement

*R*[*F* ^2^ > 2σ(*F* ^2^)] = 0.049
*wR*(*F* ^2^) = 0.152
*S* = 1.01
2705 reflections
156 parameters
H-atom parameters constrained
Δρ_max_ = 0.28 e Å^−3^
Δρ_min_ = −0.23 e Å^−3^

### 

Data collection: *CrystalClear* (Rigaku/MSC, 2008 ▶); cell refinement: *CrystalClear*; data reduction: *CrystalClear*; program(s) used to solve structure: *SHELXS97* (Sheldrick, 2008 ▶); program(s) used to refine structure: *SHELXL97* (Sheldrick, 2008 ▶); molecular graphics: *PLATON* (Spek, 2009 ▶); software used to prepare material for publication: *SHELXL97*.

## Supplementary Material

Crystal structure: contains datablocks global, I. DOI: 10.1107/S1600536810037839/cv2768sup1.cif

Structure factors: contains datablocks I. DOI: 10.1107/S1600536810037839/cv2768Isup2.hkl

Additional supplementary materials:  crystallographic information; 3D view; checkCIF report

## Figures and Tables

**Fig. 1.**
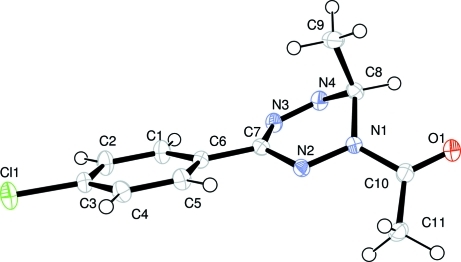
The structure of (I), shown with 30% probability displacement ellipsoids.

## supplementary crystallographic information

### Comment

1,2,4,5-Tetrazine derivatives have high potential for biological activity, possessing a wide spectrum of antiviral and antitumor properties. They have been widely used in pesticides and herbicides (Sauer,1996). Dihydro-1,2,4,5- tetrazine has four isomers, namely 1,2-, 1,4-, 1,6- and 3,6-dihydro-1,2,4,5- tetrazines. The 1,6-dihydro structures (Stam *et al.*, 1982; Jennison *et al.*, 1986) were found, by X-ray diffraction, to be homoaromatic. In continuation of our work on the structure-activity relationship of 1,6-dihydro-1,2,4,5-tetrazine derivatives (Hu *et al.*, 2004, 2005), we have obtained the title compound, (I) (Fig.1).

In the tetrazine ring of (I), atoms N1, N2, N3 and N4 are coplanar, while atoms C7 and C8 deviate from the plane by 0.254 (3) and 0.621 (3) Å, respectively. The N2/C7/N3 and N1/C8/N4 planes make dihedral angles of 7.61 (2)° and 44.04 (2)°, respectively, with the N1/N2/N3/N4 plane, the tetrazine ring adopts an unsymmetrical boat conformation. The benzene ring make dihedral angle of 18.5 (2)° with the N1/N2/N3/N4 plane. N1 is almost *sp*^2^ hybridized due to the angles around it add up to 359.9 (2)°. Compairing with similar situations in 3-phenyl-6-ethyl- 1,6-dihydro-1,2,4,5-tetrazine (Stam *et al.*, 1982), *N*-(2-methylphenyl)-3-phenyl-6-methyl-1,6-dihydro-1,2,4,5-tetrazine (Xu *et al.*,2010), 1-acetyl-3,6-dimethyl-1,2,4,5-tetrazine (Jennison *et al.*,1986) and 1-[3-(4-Methoxyphenyl)-6-methyl-1,6-dihydro-1,2,4,5- tetrazin-1-yl]propanone (Yang *et al.*, 2010), one can state that the molecule in (I) is homoaromatic.

### Experimental

3-(4-Chlorophenyl)-6-methyl-1,6-dihydro-1,2,4,5-tetrazine (3.0 mmol), chloroform (10 ml) and pyridine(0.25 ml,3.1 mmol) were mixed. Acetyl chloride(3.0 mmol) in chloroform (10 ml) was added dropwise with stirring at room temperature. After the starting 1,6-dihydro-1,2,4,5-tetrazine was completely consumed (the reaction courses was monitored by TLC,dichloromethane system), evaporation of the chloroform, crude 1-acetyl-3-(4-chlorophenyl)-6-methyl- 1,6-dihydro-1,2,4,5-tetrazine was obtained and purified by preparative thin-layer chromatography over silica gel GF254(2 mm) (dichloromethane: petroleum ether=1:1). The solution of the compound in anhydrous ethanol was concentrated gradually at room temperature to afford single crystals, which was suitable for X-ray diffraction. (*M*. P. 352–354 K).^1^H NMR (CDCl_3_) δ p.p.m.: 8.10 (d,2*H*, J = 8.0 Hz), 7.49 (d,2*H*, J = 8.0 Hz), 6.87 (q,1*H*, J = 6.7 Hz), 2.49(s,3*H*), 1.05(d,3*H*, J = 6.4 Hz).

### Refinement

Methyl H atoms were placed in calculated positions with C—H = 0.96 Å and torsion angles were refined to fit the electron density, *U*_iso_(H) = 1.5*U*_eq_(C). Other H atoms were placed in calculated positions with C—H = 0.93 and N—H = 0.86 Å, and refined in riding mode, with *U*_iso_(H) = 1.2*U*_eq_(C,*N*).

### Crystal data

**Table:**

C_11_H_11_ClN_4_O	*F*(000) = 1040
*M**_r_* = 250.69	*D*_x_ = 1.411 Mg m^−^^3^
Orthorhombic, *P**b**c**a*	Mo *K*α radiation, λ = 0.71073 Å
Hall symbol: -P 2ac 2ab	Cell parameters from 6560 reflections
*a* = 15.165 (3) Å	θ = 3.1–27.5°
*b* = 8.0452 (15) Å	µ = 0.31 mm^−^^1^
*c* = 19.349 (4) Å	*T* = 93 K
*V* = 2360.7 (8) Å^3^	Block, orange
*Z* = 8	0.47 × 0.40 × 0.37 mm

### Data collection

**Table:**

Rigaku AFC10/Saturn724+ diffractometer	2599 reflections with *I* > 2σ(*I*)
Radiation source: Rotating Anode	*R*_int_ = 0.038
graphite	θ_max_ = 27.5°, θ_min_ = 3.4°
Detector resolution: 28.5714 pixels mm^-1^	*h* = −15→19
phi and ω scans	*k* = −7→10
17617 measured reflections	*l* = −25→25
2705 independent reflections	

### Refinement

**Table:**

Refinement on *F*^2^	Primary atom site location: structure-invariant direct methods
Least-squares matrix: full	Secondary atom site location: difference Fourier map
*R*[*F*^2^ > 2σ(*F*^2^)] = 0.049	Hydrogen site location: inferred from neighbouring sites
*wR*(*F*^2^) = 0.152	H-atom parameters constrained
*S* = 1.01	*w* = 1/[σ^2^(*F*_o_^2^) + (0.1*P*)^2^ + 1.56*P*] where *P* = (*F*_o_^2^ + 2*F*_c_^2^)/3
2705 reflections	(Δ/σ)_max_ = 0.001
156 parameters	Δρ_max_ = 0.28 e Å^−^^3^
0 restraints	Δρ_min_ = −0.23 e Å^−^^3^

### Special details

**Table:**

Geometry. All e.s.d.'s (except the e.s.d. in the dihedral angle between two l.s. planes) are estimated using the full covariance matrix. The cell e.s.d.'s are taken into account individually in the estimation of e.s.d.'s in distances, angles and torsion angles; correlations between e.s.d.'s in cell parameters are only used when they are defined by crystal symmetry. An approximate (isotropic) treatment of cell e.s.d.'s is used for estimating e.s.d.'s involving l.s. planes.
Refinement. Refinement of *F*^2^ against ALL reflections. The weighted *R*-factor *wR* and goodness of fit *S* are based on *F*^2^, conventional *R*-factors *R* are based on *F*, with *F* set to zero for negative *F*^2^. The threshold expression of *F*^2^ > σ(*F*^2^) is used only for calculating *R*-factors(gt) *etc*. and is not relevant to the choice of reflections for refinement. *R*-factors based on *F*^2^ are statistically about twice as large as those based on *F*, and *R*- factors based on ALL data will be even larger.

### Fractional atomic coordinates and isotropic or equivalent isotropic displacement parameters (Å^2^)

**Table:**

	*x*	*y*	*z*	*U*_iso_*/*U*_eq_	
Cl1	0.92289 (3)	0.63612 (7)	0.27240 (2)	0.02638 (19)	
O1	0.28242 (9)	0.51349 (19)	0.42261 (8)	0.0260 (4)	
N1	0.42522 (10)	0.5869 (2)	0.41004 (8)	0.0165 (3)	
N2	0.50513 (10)	0.5506 (2)	0.38219 (8)	0.0167 (3)	
N3	0.56159 (11)	0.7128 (2)	0.47435 (8)	0.0191 (4)	
N4	0.48695 (11)	0.7527 (2)	0.49690 (9)	0.0203 (4)	
C1	0.73156 (13)	0.6887 (3)	0.41229 (10)	0.0226 (4)	
H1	0.7261	0.7262	0.4586	0.027*	
C2	0.81377 (13)	0.6872 (3)	0.38052 (10)	0.0234 (4)	
H2	0.8647	0.7229	0.4050	0.028*	
C3	0.82041 (13)	0.6332 (2)	0.31290 (10)	0.0194 (4)	
C4	0.74733 (13)	0.5800 (3)	0.27600 (9)	0.0202 (4)	
H4	0.7531	0.5428	0.2296	0.024*	
C5	0.66569 (13)	0.5822 (3)	0.30804 (10)	0.0200 (4)	
H5	0.6149	0.5472	0.2832	0.024*	
C6	0.65710 (12)	0.6353 (2)	0.37632 (10)	0.0168 (4)	
C7	0.56927 (13)	0.6386 (2)	0.40816 (10)	0.0164 (4)	
C8	0.41462 (13)	0.7456 (2)	0.44422 (10)	0.0184 (4)	
H8	0.3564	0.7475	0.4685	0.022*	
C9	0.42014 (14)	0.8936 (3)	0.39576 (12)	0.0251 (5)	
H9A	0.4757	0.8890	0.3699	0.030*	
H9B	0.4179	0.9966	0.4227	0.030*	
H9C	0.3705	0.8906	0.3634	0.030*	
C10	0.35516 (13)	0.4761 (2)	0.40099 (10)	0.0192 (4)	
C11	0.37533 (13)	0.3182 (2)	0.36325 (11)	0.0217 (4)	
H11A	0.3268	0.2393	0.3697	0.026*	
H11B	0.4300	0.2700	0.3814	0.026*	
H11C	0.3825	0.3418	0.3139	0.026*	

### Atomic displacement parameters (Å^2^)

**Table:**

	*U*^11^	*U*^22^	*U*^33^	*U*^12^	*U*^13^	*U*^23^
Cl1	0.0169 (3)	0.0416 (4)	0.0206 (3)	−0.00189 (19)	0.00381 (16)	−0.00357 (19)
O1	0.0156 (7)	0.0260 (8)	0.0363 (8)	−0.0012 (6)	0.0001 (6)	0.0006 (6)
N1	0.0153 (8)	0.0168 (8)	0.0175 (8)	0.0002 (6)	0.0009 (6)	0.0000 (6)
N2	0.0153 (8)	0.0174 (8)	0.0174 (8)	0.0010 (6)	0.0005 (6)	0.0019 (6)
N3	0.0181 (8)	0.0232 (9)	0.0158 (8)	−0.0004 (6)	0.0016 (6)	−0.0013 (6)
N4	0.0195 (8)	0.0231 (8)	0.0184 (8)	−0.0015 (7)	0.0008 (6)	−0.0024 (6)
C1	0.0195 (10)	0.0334 (11)	0.0148 (8)	−0.0025 (8)	0.0011 (7)	−0.0034 (8)
C2	0.0161 (9)	0.0357 (12)	0.0186 (9)	−0.0050 (8)	−0.0018 (7)	−0.0049 (8)
C3	0.0158 (9)	0.0237 (10)	0.0186 (9)	0.0000 (7)	0.0024 (7)	0.0008 (7)
C4	0.0200 (10)	0.0251 (10)	0.0153 (8)	0.0012 (8)	0.0005 (7)	−0.0035 (7)
C5	0.0178 (9)	0.0247 (10)	0.0175 (9)	−0.0008 (8)	−0.0015 (7)	−0.0027 (7)
C6	0.0163 (9)	0.0170 (9)	0.0172 (9)	−0.0010 (7)	0.0002 (7)	0.0005 (6)
C7	0.0170 (10)	0.0172 (9)	0.0150 (9)	0.0015 (7)	−0.0008 (6)	0.0004 (6)
C8	0.0178 (9)	0.0191 (9)	0.0184 (9)	0.0024 (7)	0.0010 (7)	−0.0025 (7)
C9	0.0264 (11)	0.0180 (10)	0.0309 (11)	0.0030 (8)	−0.0019 (8)	0.0014 (8)
C10	0.0174 (9)	0.0205 (10)	0.0198 (9)	−0.0011 (7)	−0.0027 (7)	0.0042 (7)
C11	0.0208 (10)	0.0182 (9)	0.0263 (10)	−0.0036 (8)	−0.0034 (7)	0.0007 (7)

### Geometric parameters (Å, °)

**Table:**

Cl1—C3	1.741 (2)	C4—C5	1.385 (3)
O1—C10	1.218 (2)	C4—H4	0.9500
N1—N2	1.358 (2)	C5—C6	1.395 (3)
N1—C10	1.398 (3)	C5—H5	0.9500
N1—C8	1.447 (2)	C6—C7	1.468 (3)
N2—C7	1.304 (2)	C8—C9	1.518 (3)
N3—N4	1.255 (2)	C8—H8	1.0000
N3—C7	1.418 (2)	C9—H9A	0.9800
N4—C8	1.499 (2)	C9—H9B	0.9800
C1—C2	1.390 (3)	C9—H9C	0.9800
C1—C6	1.394 (3)	C10—C11	1.497 (3)
C1—H1	0.9500	C11—H11A	0.9800
C2—C3	1.382 (3)	C11—H11B	0.9800
C2—H2	0.9500	C11—H11C	0.9800
C3—C4	1.386 (3)		
			
N2—N1—C10	119.42 (16)	N2—C7—N3	121.02 (17)
N2—N1—C8	118.06 (15)	N2—C7—C6	120.36 (17)
C10—N1—C8	122.38 (16)	N3—C7—C6	117.48 (16)
C7—N2—N1	113.33 (16)	N1—C8—N4	105.26 (15)
N4—N3—C7	119.73 (16)	N1—C8—C9	113.80 (16)
N3—N4—C8	114.46 (16)	N4—C8—C9	110.49 (16)
C2—C1—C6	120.20 (17)	N1—C8—H8	109.1
C2—C1—H1	119.9	N4—C8—H8	109.1
C6—C1—H1	119.9	C9—C8—H8	109.1
C3—C2—C1	119.11 (18)	C8—C9—H9A	109.5
C3—C2—H2	120.4	C8—C9—H9B	109.5
C1—C2—H2	120.4	H9A—C9—H9B	109.5
C2—C3—C4	121.77 (18)	C8—C9—H9C	109.5
C2—C3—Cl1	119.14 (15)	H9A—C9—H9C	109.5
C4—C3—Cl1	119.08 (15)	H9B—C9—H9C	109.5
C5—C4—C3	118.70 (17)	O1—C10—N1	119.20 (18)
C5—C4—H4	120.7	O1—C10—C11	124.23 (18)
C3—C4—H4	120.7	N1—C10—C11	116.56 (17)
C4—C5—C6	120.77 (18)	C10—C11—H11A	109.5
C4—C5—H5	119.6	C10—C11—H11B	109.5
C6—C5—H5	119.6	H11A—C11—H11B	109.5
C1—C6—C5	119.45 (17)	C10—C11—H11C	109.5
C1—C6—C7	121.32 (17)	H11A—C11—H11C	109.5
C5—C6—C7	119.21 (17)	H11B—C11—H11C	109.5
			
C10—N1—N2—C7	−163.54 (16)	N4—N3—C7—C6	163.93 (18)
C8—N1—N2—C7	20.6 (2)	C1—C6—C7—N2	−162.04 (18)
C7—N3—N4—C8	−11.1 (3)	C5—C6—C7—N2	19.9 (3)
C6—C1—C2—C3	0.4 (3)	C1—C6—C7—N3	5.9 (3)
C1—C2—C3—C4	−0.2 (3)	C5—C6—C7—N3	−172.22 (17)
C1—C2—C3—Cl1	178.44 (17)	N2—N1—C8—N4	−54.1 (2)
C2—C3—C4—C5	0.3 (3)	C10—N1—C8—N4	130.11 (17)
Cl1—C3—C4—C5	−178.33 (16)	N2—N1—C8—C9	67.0 (2)
C3—C4—C5—C6	−0.6 (3)	C10—N1—C8—C9	−108.8 (2)
C2—C1—C6—C5	−0.7 (3)	N3—N4—C8—N1	47.4 (2)
C2—C1—C6—C7	−178.79 (19)	N3—N4—C8—C9	−75.8 (2)
C4—C5—C6—C1	0.8 (3)	N2—N1—C10—O1	−175.83 (17)
C4—C5—C6—C7	178.96 (19)	C8—N1—C10—O1	−0.1 (3)
N1—N2—C7—N3	22.6 (2)	N2—N1—C10—C11	2.9 (2)
N1—N2—C7—C6	−169.92 (16)	C8—N1—C10—C11	178.63 (17)
N4—N3—C7—N2	−28.2 (3)		
